# Undeca­carbonyl-1κ^3^ *C*,2κ^4^ *C*,3κ^4^ *C*-[tris­(3-chloro­phen­yl)phosphine-1κ*P*]-*triangulo*-triruthenium(0)

**DOI:** 10.1107/S1600536810013826

**Published:** 2010-04-28

**Authors:** Omar bin Shawkataly, Mohd. Aslam A. Pankhi, Chin Sing Yeap, Hoong-Kun Fun

**Affiliations:** a Chemical Sciences Programme, School of Distance Education, Universiti Sains Malaysia, 11800 USM, Penang, Malaysia; b X-ray Crystallography Unit, School of Physics, Universiti Sains Malaysia, 11800 USM, Penang, Malaysia

## Abstract

In the title *triangulo*-triruthenium compound, [Ru_3_(C_18_H_12_Cl_3_P)(CO)_11_], one equatorial carbonyl group has been substituted by the monodentate phosphine ligand, leaving one equatorial and two axial carbonyl substituents on the Ru atom. The remaining two Ru atoms each carry two equatorial and two axial terminal carbonyl ligands. The three benzene rings make dihedral angles of 87.83 (17), 69.91 (17) and 68.26 (17)° with each other. In the crystal structure, mol­ecules are linked into dimers by inter­molecular C—H⋯O hydrogen bonds. The mol­ecular structure is stabilized by an intra­molecular C—H⋯O hydrogen bond.

## Related literature

For related structures, see: Bruce *et al.* (1988 ▶); Churchill *et al.* (1977 ▶). For the synthesis, see: Bruce *et al.* (1987 ▶). For the stability of the temperature controller used for the data collection, see: Cosier & Glazer (1986 ▶).
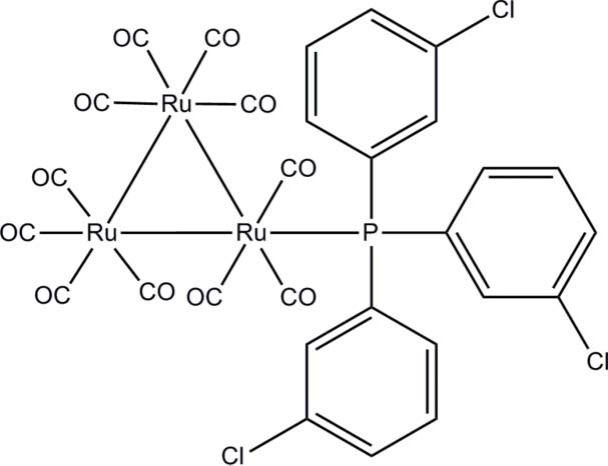


## Experimental

### 

#### Crystal data

[Ru_3_(C_18_H_12_Cl_3_P)(CO)_11_]
*M* *_r_* = 976.92
Monoclinic, 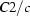

*a* = 21.8834 (6) Å
*b* = 17.1060 (5) Å
*c* = 18.4776 (5) Å
β = 107.766 (2)°
*V* = 6587.0 (3) Å^3^
*Z* = 8
Mo *K*α radiation
μ = 1.71 mm^−1^
*T* = 100 K
0.24 × 0.19 × 0.12 mm

#### Data collection

Bruker SMART APEXII CCD area-detector diffractometer
Absorption correction: multi-scan (*SADABS*; Bruker, 2009 ▶) *T*_min_ = 0.686, *T*_max_ = 0.820
39715 measured reflections
9694 independent reflections
7635 reflections with *I* > 2σ(*I*)
*R*_int_ = 0.044

#### Refinement

*R*[*F* ^2^ > 2σ(*F* ^2^)] = 0.040
*wR*(*F* ^2^) = 0.111
*S* = 1.03
9694 reflections
425 parameters
H-atom parameters constrained
Δρ_max_ = 2.26 e Å^−3^
Δρ_min_ = −1.16 e Å^−3^

### 

Data collection: *APEX2* (Bruker, 2009 ▶); cell refinement: *SAINT* (Bruker, 2009 ▶); data reduction: *SAINT*; program(s) used to solve structure: *SHELXTL* (Sheldrick, 2008 ▶); program(s) used to refine structure: *SHELXTL*; molecular graphics: *SHELXTL*; software used to prepare material for publication: *SHELXTL* and *PLATON* (Spek, 2009 ▶).

## Supplementary Material

Crystal structure: contains datablocks global, I. DOI: 10.1107/S1600536810013826/sj2762sup1.cif

Structure factors: contains datablocks I. DOI: 10.1107/S1600536810013826/sj2762Isup2.hkl

Additional supplementary materials:  crystallographic information; 3D view; checkCIF report

## Figures and Tables

**Table 1:** Hydrogen-bond geometry (Å, °)

*D*—H⋯*A*	*D*—H	H⋯*A*	*D*⋯*A*	*D*—H⋯*A*
C17—H17*A*⋯O5^i^	0.93	2.48	3.277 (5)	143
C18—H18*A*⋯O3	0.93	2.59	3.165 (5)	121

Symmetry code: (i) 
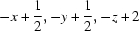
.

**Fig. 1.**
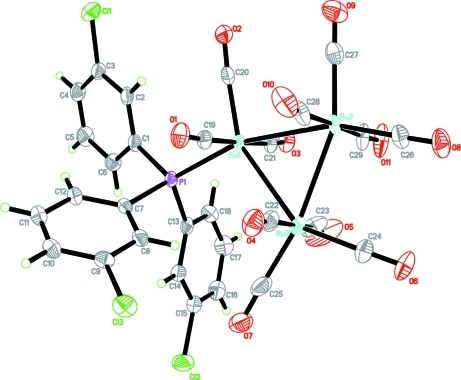
The molecular structure of the title compound with 50% probability ellipsoids for non-H atoms.

**Fig. 2.**
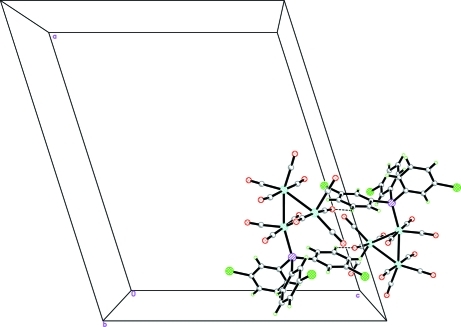
A pair of molecules is linked into a dimer by intermolecular hydrogen bonds (dashed lines).

## supplementary crystallographic information

### Comment

Syntheses and structures of substituted *triangulo*-triruthenium clusters have been of interest to researchers due to observed structural variations and their potential catalytic activity. As part of our ongoing studies on phosphine substituted *triangulo*-triruthenium clusters, herein we report the structure of title compound (I).

In the title compound (I), the monodentate phosphine ligand has replaced a single carbonyl group in the equatorial plane of the Ru_3_ triangle. The *triangulo*-triruthenium is bonded equatorially to a monodentate phosphine ligand.  The Ru1–Ru2 bond is noticeably longer [2.9002 (4) Å] compared to the other two Ru–Ru bonds [2.8600 (3) and 2.8611 (4) Å]. The unusual increase in the length of Ru–Ru bond in comparison to those in Ru_3_(CO)_12_ (Churchill *et al.*, 1977), can be attributed to the steric effect induced by the bulky substituent.

As observed in Ru_3_(CO)_12_, the bond from metal atoms to the axial CO ligands in complex (I) are longer (Ru–C(ave) =  1.934 Å) compared to the equatorial CO groups (Ru–C(ave) =  1.918 Å). The equatorial Ru–C–O substituents are linear (average value: 177.94°) while the axial Ru–C–O ligands are slightly bent (average value: 173.55°). Similar observations were made by Bruce and co-workers for the range of monosubstituted complexes they synthesized (Bruce *et al.*, 1988).

The three phosphine-substituted benzene rings make dihedral angles (C1–C6/C7–C12, C1–C6/C13–C18 and C7–C12/C13–C18) of 87.83 (17), 69.91 (17) and 68.26 (17)° with each other respectively. In the crystal structure, the molecules are linked into dimers by intermolecular C17—H17A···O5 hydrogen bonds (Fig. 2, Table 1). The molecular structure is stabilized by an intramolecular C18—H18A···O3 hydrogen bond.

### Experimental

All the manipulations were performed under a dry oxygen-free nitrogen atmosphere using standard Schlenk techniques. THF was dried over sodium wire and freshly distilled from sodium benzophenone ketyl solution. The title compound (I) was prepared by mixing Ru_3_(CO)_12_ (Aldrich) and P(3-Cl-C_6_H_4_)_3_ (Maybridge) in a 1:1 molar ratio in THF at 40 °C. About 0.2 ml of diphenylketyl radical anion initiator (synthesized as per the method of Bruce *et al.*,  1987) was introduced into the reaction mixture under a current of nitrogen. After 15 min. of stirring, the solvent was removed under vacuum. Separation of the product in the pure form was done by column chromatography (Florisil, 100-200 mesh, eluant, dichloromethane: hexane). IR (cyclohexane): ν (CO) 2100, 2049, 2033 and 2019 cm^-1^. ^1^H-NMR (CDCl_3_, δ); 7.23-7.25 (m, aromatic protons). Crystals suitable for X-ray diffraction were grown from n-pentane solution at 10°C.

### Refinement

All hydrogen atoms were positioned geometrically and refined using a riding model with C—H = 0.93 Å and *U*_iso_(H) = 1.2 *U*_eq_(C).

### Crystal data

**Table:**

[Ru_3_(C_18_H_12_Cl_3_P)(CO)_11_]	*F*(000) = 3776
*M**_r_* = 976.92	*D*_x_ = 1.970 Mg m^−^^3^
Monoclinic, *C*2/*c*	Mo *K*α radiation, λ = 0.71073 Å
Hall symbol: -C 2yc	Cell parameters from 9924 reflections
*a* = 21.8834 (6) Å	θ = 2.5–30.1°
*b* = 17.1060 (5) Å	µ = 1.71 mm^−^^1^
*c* = 18.4776 (5) Å	*T* = 100 K
β = 107.766 (2)°	Block, orange
*V* = 6587.0 (3) Å^3^	0.24 × 0.19 × 0.12 mm
*Z* = 8	

### Data collection

**Table:**

Bruker SMART APEXII CCD area-detector diffractometer	9694 independent reflections
Radiation source: fine-focus sealed tube	7635 reflections with *I* > 2σ(*I*)
graphite	*R*_int_ = 0.044
φ and ω scans	θ_max_ = 30.2°, θ_min_ = 2.0°
Absorption correction: multi-scan (*SADABS*; Bruker, 2009)	*h* = −30→30
*T*_min_ = 0.686, *T*_max_ = 0.820	*k* = −21→24
39715 measured reflections	*l* = −26→25

### Refinement

**Table:**

Refinement on *F*^2^	Primary atom site location: structure-invariant direct methods
Least-squares matrix: full	Secondary atom site location: difference Fourier map
*R*[*F*^2^ > 2σ(*F*^2^)] = 0.040	Hydrogen site location: inferred from neighbouring sites
*wR*(*F*^2^) = 0.111	H-atom parameters constrained
*S* = 1.03	*w* = 1/[σ^2^(*F*_o_^2^) + (0.0605*P*)^2^ + 18.2026*P*] where *P* = (*F*_o_^2^ + 2*F*_c_^2^)/3
9694 reflections	(Δ/σ)_max_ = 0.002
425 parameters	Δρ_max_ = 2.26 e Å^−^^3^
0 restraints	Δρ_min_ = −1.16 e Å^−^^3^

### Special details

**Table:**

Experimental. The crystal was placed in the cold stream of an Oxford Cryosystems Cobra open-flow nitrogen cryostat (Cosier & Glazer, 1986) operating at 100.0 (1) K.
Geometry. All esds (except the esd in the dihedral angle between two l.s. planes) are estimated using the full covariance matrix. The cell esds are taken into account individually in the estimation of esds in distances, angles and torsion angles; correlations between esds in cell parameters are only used when they are defined by crystal symmetry. An approximate (isotropic) treatment of cell esds is used for estimating esds involving l.s. planes.
Refinement. Refinement of *F*^2^ against ALL reflections. The weighted *R*-factor wR and goodness of fit *S* are based on *F*^2^, conventional *R*-factors *R* are based on *F*, with *F* set to zero for negative *F*^2^. The threshold expression of *F*^2^ > σ(*F*^2^) is used only for calculating *R*-factors(gt) etc. and is not relevant to the choice of reflections for refinement. *R*-factors based on *F*^2^ are statistically about twice as large as those based on *F*, and *R*- factors based on ALL data will be even larger.

### Fractional atomic coordinates and isotropic or equivalent isotropic displacement parameters (Å^2^)

**Table:**

	*x*	*y*	*z*	*U*_iso_*/*U*_eq_	
Ru1	0.265976 (11)	0.441176 (16)	0.750536 (14)	0.01723 (7)	
Ru2	0.320122 (13)	0.531715 (18)	0.887920 (15)	0.02321 (8)	
Ru3	0.398579 (12)	0.484230 (17)	0.797051 (16)	0.02177 (7)	
Cl1	0.09752 (5)	0.27671 (6)	0.46981 (5)	0.0353 (2)	
Cl2	0.09334 (6)	0.46270 (8)	1.01036 (6)	0.0443 (3)	
Cl3	0.12225 (5)	0.73140 (6)	0.79389 (6)	0.03193 (19)	
P1	0.15807 (4)	0.42273 (5)	0.74605 (5)	0.01757 (16)	
O1	0.22735 (13)	0.59340 (17)	0.65962 (15)	0.0311 (6)	
O2	0.27915 (13)	0.36091 (19)	0.60988 (15)	0.0372 (7)	
O3	0.30269 (12)	0.28275 (16)	0.83071 (15)	0.0291 (6)	
O4	0.29618 (14)	0.68820 (18)	0.80133 (16)	0.0373 (7)	
O5	0.3271 (2)	0.3759 (2)	0.9712 (2)	0.0693 (13)	
O6	0.44161 (14)	0.5858 (2)	1.00971 (16)	0.0422 (7)	
O7	0.21662 (16)	0.5775 (3)	0.95945 (19)	0.0553 (10)	
O8	0.52487 (13)	0.56379 (18)	0.88341 (19)	0.0401 (7)	
O9	0.43184 (15)	0.39590 (19)	0.67010 (19)	0.0409 (7)	
O10	0.36786 (15)	0.62871 (19)	0.6937 (2)	0.0435 (8)	
O11	0.43524 (14)	0.33874 (19)	0.89892 (19)	0.0471 (9)	
C1	0.11003 (15)	0.3518 (2)	0.67796 (19)	0.0212 (6)	
C2	0.12037 (15)	0.3422 (2)	0.60809 (19)	0.0236 (7)	
H2A	0.1532	0.3695	0.5970	0.028*	
C3	0.08139 (16)	0.2915 (2)	0.55467 (19)	0.0257 (7)	
C4	0.03140 (17)	0.2512 (2)	0.5694 (2)	0.0289 (8)	
H4A	0.0054	0.2180	0.5331	0.035*	
C5	0.02093 (17)	0.2612 (2)	0.6382 (2)	0.0312 (8)	
H5A	−0.0125	0.2345	0.6485	0.037*	
C6	0.05988 (16)	0.3110 (2)	0.6931 (2)	0.0267 (7)	
H6A	0.0524	0.3171	0.7397	0.032*	
C7	0.10694 (15)	0.5099 (2)	0.72235 (18)	0.0194 (6)	
C8	0.12995 (15)	0.5795 (2)	0.76087 (19)	0.0229 (7)	
H8A	0.1693	0.5802	0.7988	0.028*	
C9	0.09408 (16)	0.6470 (2)	0.7424 (2)	0.0234 (7)	
C10	0.03585 (17)	0.6488 (2)	0.6848 (2)	0.0252 (7)	
H10A	0.0127	0.6950	0.6722	0.030*	
C11	0.01323 (16)	0.5797 (2)	0.6467 (2)	0.0252 (7)	
H11A	−0.0256	0.5798	0.6078	0.030*	
C12	0.04753 (15)	0.5103 (2)	0.66568 (19)	0.0214 (6)	
H12A	0.0310	0.4642	0.6407	0.026*	
C13	0.14856 (14)	0.3873 (2)	0.83526 (18)	0.0200 (6)	
C14	0.12485 (16)	0.4338 (2)	0.88253 (19)	0.0233 (7)	
H14A	0.1102	0.4841	0.8676	0.028*	
C15	0.12338 (17)	0.4044 (2)	0.9522 (2)	0.0278 (7)	
C16	0.14433 (18)	0.3296 (2)	0.9762 (2)	0.0315 (8)	
H16A	0.1435	0.3111	1.0232	0.038*	
C17	0.16670 (19)	0.2831 (2)	0.9279 (2)	0.0320 (8)	
H17A	0.1806	0.2325	0.9426	0.038*	
C18	0.16863 (16)	0.3109 (2)	0.8584 (2)	0.0252 (7)	
H18A	0.1834	0.2788	0.8267	0.030*	
C19	0.24350 (15)	0.5393 (2)	0.69618 (19)	0.0227 (7)	
C20	0.27195 (15)	0.3914 (2)	0.66209 (19)	0.0242 (7)	
C21	0.29008 (15)	0.3434 (2)	0.80467 (19)	0.0236 (7)	
C22	0.30548 (17)	0.6282 (2)	0.8299 (2)	0.0277 (7)	
C23	0.3247 (2)	0.4307 (3)	0.9355 (2)	0.0413 (11)	
C24	0.39645 (18)	0.5678 (2)	0.9644 (2)	0.0308 (8)	
C25	0.2546 (2)	0.5596 (3)	0.9320 (2)	0.0382 (10)	
C26	0.47775 (17)	0.5337 (2)	0.8521 (2)	0.0297 (8)	
C27	0.41920 (17)	0.4287 (2)	0.7170 (2)	0.0281 (8)	
C28	0.37533 (16)	0.5754 (2)	0.7328 (2)	0.0293 (8)	
C29	0.41894 (17)	0.3925 (2)	0.8626 (2)	0.0331 (9)	

### Atomic displacement parameters (Å^2^)

**Table:**

	*U*^11^	*U*^22^	*U*^33^	*U*^12^	*U*^13^	*U*^23^
Ru1	0.01205 (11)	0.01875 (14)	0.01862 (12)	−0.00079 (9)	0.00130 (8)	−0.00045 (9)
Ru2	0.02123 (13)	0.02513 (16)	0.02064 (13)	−0.00779 (10)	0.00248 (10)	−0.00305 (10)
Ru3	0.01236 (11)	0.01986 (15)	0.03029 (14)	−0.00061 (9)	0.00233 (9)	0.00255 (10)
Cl1	0.0368 (5)	0.0417 (6)	0.0245 (4)	−0.0026 (4)	0.0050 (3)	−0.0055 (4)
Cl2	0.0517 (6)	0.0592 (7)	0.0245 (4)	0.0184 (5)	0.0156 (4)	0.0018 (4)
Cl3	0.0349 (4)	0.0196 (4)	0.0422 (5)	−0.0040 (4)	0.0131 (4)	−0.0062 (3)
P1	0.0128 (3)	0.0178 (4)	0.0202 (4)	−0.0014 (3)	0.0022 (3)	−0.0025 (3)
O1	0.0281 (12)	0.0282 (15)	0.0317 (13)	0.0005 (11)	0.0013 (10)	0.0076 (11)
O2	0.0290 (13)	0.051 (2)	0.0274 (13)	0.0147 (13)	0.0030 (11)	−0.0069 (12)
O3	0.0272 (12)	0.0228 (14)	0.0327 (13)	−0.0011 (10)	0.0023 (10)	0.0036 (10)
O4	0.0382 (15)	0.0279 (16)	0.0378 (15)	0.0029 (12)	−0.0002 (12)	0.0003 (12)
O5	0.133 (4)	0.038 (2)	0.0383 (18)	−0.027 (2)	0.028 (2)	0.0040 (15)
O6	0.0384 (15)	0.0443 (19)	0.0321 (14)	−0.0141 (14)	−0.0070 (12)	−0.0015 (13)
O7	0.0343 (16)	0.091 (3)	0.0429 (18)	−0.0146 (17)	0.0154 (14)	−0.0321 (18)
O8	0.0219 (12)	0.0332 (17)	0.060 (2)	−0.0075 (11)	0.0048 (12)	−0.0073 (14)
O9	0.0397 (16)	0.0364 (18)	0.0541 (19)	−0.0133 (13)	0.0256 (14)	−0.0115 (14)
O10	0.0369 (16)	0.0356 (18)	0.063 (2)	0.0065 (13)	0.0220 (15)	0.0194 (15)
O11	0.0288 (14)	0.0358 (18)	0.0574 (19)	−0.0068 (12)	−0.0157 (13)	0.0203 (14)
C1	0.0157 (13)	0.0187 (17)	0.0253 (15)	0.0003 (12)	0.0005 (11)	−0.0039 (12)
C2	0.0158 (13)	0.0256 (19)	0.0250 (15)	0.0034 (12)	−0.0004 (12)	−0.0019 (13)
C3	0.0209 (15)	0.0282 (19)	0.0234 (15)	0.0029 (13)	−0.0001 (12)	−0.0058 (13)
C4	0.0227 (15)	0.027 (2)	0.0317 (18)	−0.0005 (14)	0.0000 (13)	−0.0084 (14)
C5	0.0224 (16)	0.028 (2)	0.040 (2)	−0.0093 (14)	0.0049 (14)	−0.0079 (15)
C6	0.0201 (15)	0.027 (2)	0.0312 (17)	−0.0053 (14)	0.0053 (13)	−0.0069 (14)
C7	0.0164 (13)	0.0214 (17)	0.0214 (14)	0.0010 (12)	0.0072 (11)	−0.0004 (12)
C8	0.0169 (13)	0.0260 (19)	0.0261 (16)	−0.0005 (13)	0.0070 (12)	−0.0037 (13)
C9	0.0259 (16)	0.0190 (17)	0.0280 (16)	−0.0026 (13)	0.0123 (13)	−0.0030 (12)
C10	0.0236 (15)	0.0222 (18)	0.0306 (17)	0.0040 (13)	0.0096 (13)	0.0031 (13)
C11	0.0210 (14)	0.0252 (19)	0.0289 (17)	0.0032 (13)	0.0070 (13)	0.0029 (13)
C12	0.0171 (13)	0.0209 (17)	0.0256 (15)	0.0008 (12)	0.0054 (12)	0.0010 (12)
C13	0.0139 (12)	0.0226 (17)	0.0210 (14)	−0.0063 (12)	0.0018 (11)	−0.0009 (11)
C14	0.0200 (14)	0.0259 (19)	0.0225 (15)	0.0003 (13)	0.0043 (12)	0.0001 (12)
C15	0.0216 (15)	0.036 (2)	0.0237 (16)	0.0020 (14)	0.0038 (12)	−0.0016 (14)
C16	0.0270 (17)	0.037 (2)	0.0294 (18)	−0.0038 (16)	0.0073 (14)	0.0067 (15)
C17	0.0328 (18)	0.027 (2)	0.037 (2)	−0.0033 (15)	0.0115 (15)	0.0065 (15)
C18	0.0242 (15)	0.0197 (18)	0.0329 (18)	−0.0027 (13)	0.0107 (13)	−0.0015 (13)
C19	0.0162 (13)	0.0268 (19)	0.0234 (15)	−0.0040 (13)	0.0036 (12)	−0.0023 (13)
C20	0.0144 (13)	0.031 (2)	0.0256 (16)	0.0036 (13)	0.0033 (12)	0.0021 (13)
C21	0.0154 (13)	0.028 (2)	0.0246 (16)	−0.0047 (13)	0.0023 (11)	−0.0031 (13)
C22	0.0233 (15)	0.029 (2)	0.0263 (16)	−0.0028 (14)	0.0015 (13)	−0.0057 (14)
C23	0.063 (3)	0.034 (2)	0.0273 (19)	−0.017 (2)	0.0127 (19)	−0.0054 (16)
C24	0.0278 (17)	0.029 (2)	0.0305 (18)	−0.0052 (15)	0.0019 (14)	0.0006 (14)
C25	0.034 (2)	0.052 (3)	0.0261 (18)	−0.0146 (19)	0.0058 (15)	−0.0151 (17)
C26	0.0206 (15)	0.025 (2)	0.041 (2)	−0.0007 (14)	0.0067 (14)	−0.0013 (15)
C27	0.0210 (15)	0.0245 (19)	0.038 (2)	−0.0061 (14)	0.0082 (14)	0.0025 (15)
C28	0.0168 (14)	0.027 (2)	0.045 (2)	0.0006 (14)	0.0097 (14)	0.0049 (16)
C29	0.0184 (15)	0.029 (2)	0.041 (2)	−0.0057 (14)	−0.0067 (14)	0.0055 (16)

### Geometric parameters (Å, °)

**Table:**

Ru1—C20	1.882 (4)	O11—C29	1.130 (5)
Ru1—C21	1.938 (4)	C1—C2	1.387 (5)
Ru1—C19	1.941 (4)	C1—C6	1.398 (5)
Ru1—P1	2.3587 (8)	C2—C3	1.393 (5)
Ru1—Ru3	2.8600 (3)	C2—H2A	0.9300
Ru1—Ru2	2.9002 (4)	C3—C4	1.388 (5)
Ru2—C25	1.912 (4)	C4—C5	1.369 (5)
Ru2—C23	1.928 (5)	C4—H4A	0.9300
Ru2—C24	1.930 (4)	C5—C6	1.399 (5)
Ru2—C22	1.941 (4)	C5—H5A	0.9300
Ru2—Ru3	2.8611 (4)	C6—H6A	0.9300
Ru3—C26	1.918 (4)	C7—C12	1.399 (4)
Ru3—C27	1.923 (4)	C7—C8	1.399 (5)
Ru3—C28	1.932 (4)	C8—C9	1.380 (5)
Ru3—C29	1.949 (4)	C8—H8A	0.9300
Cl1—C3	1.728 (4)	C9—C10	1.388 (5)
Cl2—C15	1.735 (4)	C10—C11	1.388 (5)
Cl3—C9	1.736 (4)	C10—H10A	0.9300
P1—C13	1.827 (3)	C11—C12	1.391 (5)
P1—C1	1.832 (3)	C11—H11A	0.9300
P1—C7	1.836 (3)	C12—H12A	0.9300
O1—C19	1.136 (4)	C13—C14	1.394 (5)
O2—C20	1.149 (4)	C13—C18	1.402 (5)
O3—C21	1.141 (4)	C14—C15	1.393 (5)
O4—C22	1.143 (5)	C14—H14A	0.9300
O5—C23	1.138 (5)	C15—C16	1.385 (6)
O6—C24	1.126 (4)	C16—C17	1.392 (6)
O7—C25	1.140 (5)	C16—H16A	0.9300
O8—C26	1.140 (4)	C17—C18	1.382 (5)
O9—C27	1.136 (5)	C17—H17A	0.9300
O10—C28	1.144 (5)	C18—H18A	0.9300
			
C20—Ru1—C21	88.73 (15)	C4—C3—Cl1	119.8 (3)
C20—Ru1—C19	90.89 (15)	C2—C3—Cl1	118.9 (3)
C21—Ru1—C19	178.89 (14)	C5—C4—C3	119.0 (3)
C20—Ru1—P1	104.07 (10)	C5—C4—H4A	120.5
C21—Ru1—P1	90.84 (10)	C3—C4—H4A	120.5
C19—Ru1—P1	90.27 (10)	C4—C5—C6	120.8 (4)
C20—Ru1—Ru3	92.55 (10)	C4—C5—H5A	119.6
C21—Ru1—Ru3	88.61 (9)	C6—C5—H5A	119.6
C19—Ru1—Ru3	90.36 (9)	C1—C6—C5	120.0 (3)
P1—Ru1—Ru3	163.36 (2)	C1—C6—H6A	120.0
C20—Ru1—Ru2	152.05 (10)	C5—C6—H6A	120.0
C21—Ru1—Ru2	92.01 (10)	C12—C7—C8	119.0 (3)
C19—Ru1—Ru2	87.84 (10)	C12—C7—P1	122.8 (3)
P1—Ru1—Ru2	103.85 (2)	C8—C7—P1	118.1 (2)
Ru3—Ru1—Ru2	59.559 (9)	C9—C8—C7	119.8 (3)
C25—Ru2—C23	88.3 (2)	C9—C8—H8A	120.1
C25—Ru2—C24	101.67 (17)	C7—C8—H8A	120.1
C23—Ru2—C24	92.28 (18)	C8—C9—C10	121.9 (3)
C25—Ru2—C22	90.27 (19)	C8—C9—Cl3	118.7 (3)
C23—Ru2—C22	172.41 (17)	C10—C9—Cl3	119.4 (3)
C24—Ru2—C22	95.30 (16)	C11—C10—C9	118.1 (3)
C25—Ru2—Ru3	169.30 (11)	C11—C10—H10A	120.9
C23—Ru2—Ru3	93.22 (15)	C9—C10—H10A	120.9
C24—Ru2—Ru3	88.86 (13)	C10—C11—C12	121.2 (3)
C22—Ru2—Ru3	86.82 (11)	C10—C11—H11A	119.4
C25—Ru2—Ru1	110.29 (11)	C12—C11—H11A	119.4
C23—Ru2—Ru1	82.60 (12)	C11—C12—C7	120.0 (3)
C24—Ru2—Ru1	147.40 (13)	C11—C12—H12A	120.0
C22—Ru2—Ru1	90.92 (10)	C7—C12—H12A	120.0
Ru3—Ru2—Ru1	59.521 (9)	C14—C13—C18	118.9 (3)
C26—Ru3—C27	103.89 (16)	C14—C13—P1	122.8 (3)
C26—Ru3—C28	89.76 (16)	C18—C13—P1	118.2 (3)
C27—Ru3—C28	90.31 (17)	C15—C14—C13	119.4 (3)
C26—Ru3—C29	91.55 (16)	C15—C14—H14A	120.3
C27—Ru3—C29	91.02 (18)	C13—C14—H14A	120.3
C28—Ru3—C29	177.85 (16)	C16—C15—C14	122.0 (4)
C26—Ru3—Ru1	160.49 (12)	C16—C15—Cl2	119.0 (3)
C27—Ru3—Ru1	95.53 (10)	C14—C15—Cl2	119.0 (3)
C28—Ru3—Ru1	88.31 (10)	C15—C16—C17	118.1 (4)
C29—Ru3—Ru1	89.89 (10)	C15—C16—H16A	121.0
C26—Ru3—Ru2	99.78 (12)	C17—C16—H16A	121.0
C27—Ru3—Ru2	156.20 (10)	C18—C17—C16	121.0 (4)
C28—Ru3—Ru2	92.22 (12)	C18—C17—H17A	119.5
C29—Ru3—Ru2	85.88 (13)	C16—C17—H17A	119.5
Ru1—Ru3—Ru2	60.919 (9)	C17—C18—C13	120.6 (3)
C13—P1—C1	101.54 (15)	C17—C18—H18A	119.7
C13—P1—C7	104.84 (15)	C13—C18—H18A	119.7
C1—P1—C7	101.22 (15)	O1—C19—Ru1	174.5 (3)
C13—P1—Ru1	113.65 (10)	O2—C20—Ru1	176.3 (3)
C1—P1—Ru1	118.13 (11)	O3—C21—Ru1	174.2 (3)
C7—P1—Ru1	115.50 (11)	O4—C22—Ru2	173.9 (3)
C2—C1—C6	119.2 (3)	O5—C23—Ru2	171.7 (4)
C2—C1—P1	119.7 (3)	O6—C24—Ru2	177.2 (4)
C6—C1—P1	120.9 (3)	O7—C25—Ru2	178.2 (4)
C1—C2—C3	119.6 (3)	O8—C26—Ru3	178.5 (4)
C1—C2—H2A	120.2	O9—C27—Ru3	179.5 (4)
C3—C2—H2A	120.2	O10—C28—Ru3	172.7 (3)
C4—C3—C2	121.3 (3)	O11—C29—Ru3	174.3 (4)
			
C20—Ru1—Ru2—C25	172.5 (3)	C25—Ru2—Ru3—Ru1	−18.5 (8)
C21—Ru1—Ru2—C25	−96.40 (19)	C23—Ru2—Ru3—Ru1	79.46 (13)
C19—Ru1—Ru2—C25	84.70 (19)	C24—Ru2—Ru3—Ru1	171.68 (12)
P1—Ru1—Ru2—C25	−5.04 (16)	C22—Ru2—Ru3—Ru1	−92.94 (10)
Ru3—Ru1—Ru2—C25	176.39 (16)	C20—Ru1—P1—C13	131.87 (17)
C20—Ru1—Ru2—C23	−102.0 (3)	C21—Ru1—P1—C13	42.97 (16)
C21—Ru1—Ru2—C23	−10.97 (19)	C19—Ru1—P1—C13	−137.15 (16)
C19—Ru1—Ru2—C23	170.13 (19)	Ru3—Ru1—P1—C13	−44.98 (16)
P1—Ru1—Ru2—C23	80.39 (16)	Ru2—Ru1—P1—C13	−49.31 (13)
Ru3—Ru1—Ru2—C23	−98.18 (16)	C20—Ru1—P1—C1	13.06 (17)
C20—Ru1—Ru2—C24	−19.4 (3)	C21—Ru1—P1—C1	−75.83 (16)
C21—Ru1—Ru2—C24	71.6 (2)	C19—Ru1—P1—C1	104.05 (16)
C19—Ru1—Ru2—C24	−107.3 (2)	Ru3—Ru1—P1—C1	−163.78 (13)
P1—Ru1—Ru2—C24	163.0 (2)	Ru2—Ru1—P1—C1	−168.11 (13)
Ru3—Ru1—Ru2—C24	−15.6 (2)	C20—Ru1—P1—C7	−106.90 (16)
C20—Ru1—Ru2—C22	81.9 (3)	C21—Ru1—P1—C7	164.21 (15)
C21—Ru1—Ru2—C22	172.98 (15)	C19—Ru1—P1—C7	−15.91 (15)
C19—Ru1—Ru2—C22	−5.93 (15)	Ru3—Ru1—P1—C7	76.26 (15)
P1—Ru1—Ru2—C22	−95.67 (11)	Ru2—Ru1—P1—C7	71.93 (12)
Ru3—Ru1—Ru2—C22	85.77 (11)	C13—P1—C1—C2	−159.5 (3)
C20—Ru1—Ru2—Ru3	−3.9 (2)	C7—P1—C1—C2	92.6 (3)
C21—Ru1—Ru2—Ru3	87.21 (10)	Ru1—P1—C1—C2	−34.6 (3)
C19—Ru1—Ru2—Ru3	−91.69 (10)	C13—P1—C1—C6	25.1 (3)
P1—Ru1—Ru2—Ru3	178.56 (3)	C7—P1—C1—C6	−82.8 (3)
C20—Ru1—Ru3—C26	169.2 (4)	Ru1—P1—C1—C6	150.1 (3)
C21—Ru1—Ru3—C26	−102.1 (4)	C6—C1—C2—C3	−0.9 (5)
C19—Ru1—Ru3—C26	78.3 (4)	P1—C1—C2—C3	−176.3 (3)
P1—Ru1—Ru3—C26	−13.9 (4)	C1—C2—C3—C4	1.2 (5)
Ru2—Ru1—Ru3—C26	−9.0 (4)	C1—C2—C3—Cl1	−176.9 (3)
C20—Ru1—Ru3—C27	−5.45 (16)	C2—C3—C4—C5	−0.7 (6)
C21—Ru1—Ru3—C27	83.22 (15)	Cl1—C3—C4—C5	177.3 (3)
C19—Ru1—Ru3—C27	−96.36 (15)	C3—C4—C5—C6	−0.1 (6)
P1—Ru1—Ru3—C27	171.49 (14)	C2—C1—C6—C5	0.2 (5)
Ru2—Ru1—Ru3—C27	176.36 (11)	P1—C1—C6—C5	175.5 (3)
C20—Ru1—Ru3—C28	84.71 (17)	C4—C5—C6—C1	0.3 (6)
C21—Ru1—Ru3—C28	173.37 (16)	C13—P1—C7—C12	−104.4 (3)
C19—Ru1—Ru3—C28	−6.20 (16)	C1—P1—C7—C12	0.8 (3)
P1—Ru1—Ru3—C28	−98.35 (15)	Ru1—P1—C7—C12	129.7 (3)
Ru2—Ru1—Ru3—C28	−93.48 (13)	C13—P1—C7—C8	78.2 (3)
C20—Ru1—Ru3—C29	−96.46 (17)	C1—P1—C7—C8	−176.6 (3)
C21—Ru1—Ru3—C29	−7.80 (17)	Ru1—P1—C7—C8	−47.7 (3)
C19—Ru1—Ru3—C29	172.63 (17)	C12—C7—C8—C9	−0.2 (5)
P1—Ru1—Ru3—C29	80.48 (16)	P1—C7—C8—C9	177.3 (3)
Ru2—Ru1—Ru3—C29	85.35 (13)	C7—C8—C9—C10	−1.5 (5)
C20—Ru1—Ru3—Ru2	178.19 (11)	C7—C8—C9—Cl3	177.3 (3)
C21—Ru1—Ru3—Ru2	−93.15 (10)	C8—C9—C10—C11	1.5 (5)
C19—Ru1—Ru3—Ru2	87.28 (10)	Cl3—C9—C10—C11	−177.3 (3)
P1—Ru1—Ru3—Ru2	−4.87 (9)	C9—C10—C11—C12	0.4 (5)
C25—Ru2—Ru3—C26	158.4 (8)	C10—C11—C12—C7	−2.1 (5)
C23—Ru2—Ru3—C26	−103.57 (18)	C8—C7—C12—C11	2.0 (5)
C24—Ru2—Ru3—C26	−11.35 (17)	P1—C7—C12—C11	−175.4 (3)
C22—Ru2—Ru3—C26	84.02 (16)	C1—P1—C13—C14	−123.6 (3)
Ru1—Ru2—Ru3—C26	176.96 (12)	C7—P1—C13—C14	−18.5 (3)
C25—Ru2—Ru3—C27	−27.5 (9)	Ru1—P1—C13—C14	108.5 (3)
C23—Ru2—Ru3—C27	70.5 (3)	C1—P1—C13—C18	59.2 (3)
C24—Ru2—Ru3—C27	162.7 (3)	C7—P1—C13—C18	164.3 (2)
C22—Ru2—Ru3—C27	−101.9 (3)	Ru1—P1—C13—C18	−68.7 (3)
Ru1—Ru2—Ru3—C27	−9.0 (3)	C18—C13—C14—C15	1.8 (5)
C25—Ru2—Ru3—C28	68.3 (8)	P1—C13—C14—C15	−175.4 (3)
C23—Ru2—Ru3—C28	166.29 (18)	C13—C14—C15—C16	−0.5 (5)
C24—Ru2—Ru3—C28	−101.49 (17)	C13—C14—C15—Cl2	−179.6 (3)
C22—Ru2—Ru3—C28	−6.11 (15)	C14—C15—C16—C17	−0.7 (6)
Ru1—Ru2—Ru3—C28	86.83 (11)	Cl2—C15—C16—C17	178.4 (3)
C25—Ru2—Ru3—C29	−110.7 (8)	C15—C16—C17—C18	0.7 (6)
C23—Ru2—Ru3—C29	−12.70 (17)	C16—C17—C18—C13	0.6 (6)
C24—Ru2—Ru3—C29	79.52 (16)	C14—C13—C18—C17	−1.9 (5)
C22—Ru2—Ru3—C29	174.89 (14)	P1—C13—C18—C17	175.5 (3)
Ru1—Ru2—Ru3—C29	−92.17 (10)		

### Hydrogen-bond geometry (Å, °)

**Table:**

*D*—H···*A*	*D*—H	H···*A*	*D*···*A*	*D*—H···*A*
C17—H17A···O5^i^	0.93	2.48	3.277 (5)	143
C18—H18A···O3	0.93	2.59	3.165 (5)	121

Symmetry codes: (i) −*x*+1/2, −*y*+1/2, −*z*+2.
